# Dental implant status in elderly individuals requiring domiciliary dental care in Japan

**DOI:** 10.1186/s40729-021-00340-z

**Published:** 2021-04-30

**Authors:** Yoshiyuki Hagiwara, Tetsuo Ohyama, Hiroyasu Yasuda, Keisuke Seki, Takayuki Ikeda

**Affiliations:** 1 Department of Fixed Prosthodontics, Nihon University School of Dentistry, 1-8-13 Kandasurugadai, Chiyoda-ku, Tokyo, 101-8310 Japan; 2 Department of Partial Denture Prosthodontics, Nihon University School of Dentistry, 1-8-13 Kandasurugadai, Chiyoda-ku, Tokyo, 101-8310 Japan; 3 Department of Comprehensive Dentistry and Clinical Education, Nihon University School of Dentistry, 1-8-13 Kandasurugadai, Chiyoda-ku, Tokyo, 101-8310 Japan; 4 Department of Complete Denture Prosthodontics, Nihon University School of Dentistry, 1-8-13 Kandasurugadai, Chiyoda-ku, Tokyo, 101-8310 Japan

**Keywords:** Dental implant, Domiciliary dental care, Questionnaire, Elderly individuals, Complications

## Abstract

**Background:**

The presence of implants is a significant burden not only for dentists but also for caregivers and families of elderly individuals requiring nursing and domiciliary dental care. However, few reports have assessed the status of domiciliary dental care or measures employed to deal with related issues. Hence, we aimed to evaluate the dental implant status in elderly patients requiring nursing and domiciliary dental care and to determine the suitable measures for overcoming the associated limitations. A questionnaire was mailed to 1000 dentists who provided domiciliary dental care in the Tokyo metropolitan area of Japan. The questions were classified into three categories: basic information of the dentists, actual implant status of patients requiring domiciliary dental care, and implants in an aging society.

**Results:**

The response rate was 36.5%. Approximately 2% of patients requiring domiciliary dental care were implant patients. Many implant-related problems were associated with insufficiency or difficulty in cleaning around the implant, resulting in peri-implantitis. Prosthetic and more serious complications such as implant body fracture or loss were reported and frequently managed by routine follow-ups, cleaning the area around the implant, scaling and polishing, and/or pharmacological modalities. Oral care mainly involved simple toothbrushing instructions, which was not adequate.

**Conclusions:**

Our findings suggest the necessity of simplifying the oral environment and making oral care a simple task before aging individuals require nursing and domiciliary dental care.

## Background

Implant treatment has shown long-term success by regular follow-ups of patients in a dental office. Recently, there has been an increase in the number of implant patients who cannot visit a dental clinic for follow-up appointments due to their increasing age. The Japanese population has the highest life expectancy worldwide, and in 2007, Japan was declared the world’s first “super-aging” society [1]. In 2017, the percentage of elderly individuals (≥65 years, WHO definition [2]) in Japan was 23.8%, while the percentages of individuals aged 65–74 years and ≥75 years who required care were 4.2% and 29.2%, respectively [3].

Common reasons for the need of care at home or facilities for elderly individuals include advanced age, cerebrovascular disease, and dementia. These conditions affect not only the quality of oral care provided to patients but also their general health [4–7]. Costa et al. [8] reported an increase in the incidence of peri-implantitis owing to the inadequate care of implants in patients who are unable to maintain oral hygiene. Visser et al. [9, 10] also reported implant-related problems in patients with dementia. However, these articles do not report unilaterally denied implant treatments in the elderly and state that the use of implant prostheses in the elderly has contributed significantly to improving masticatory function and quality of life [4–10]. Although, in the cases of elderly people who need long-term care, they emphasize concerns about poor oral hygiene and peri-implantitis. Consequently, there is an increase in the need for domiciliary dental care [11–13] whereby dentists or dental hygienists provide dental treatment and specialized oral care by visiting the homes, care facilities, or hospitals of patients who cannot visit dental clinics for physical or psychological reasons. However, the visited location is usually not equipped with dental infrastructure; hence, the treatment provided primarily includes simple caries treatment, adjustment and repair of dentures, and oral care. However, global awareness about domiciliary dental care is inadequate, and the system differs according to the medical insurance system, number of dentists, geographical requirements, and patient populations in different countries and regions [11–13].

The oral condition of patients requiring domiciliary dental care is generally poor, and there are various limitations in the dental equipment that are used for this mode of treatment. These patients need treatment in an environment that is completely different from that in a dental clinic; therefore, the presence of implants becomes a significant burden not only for dentists but also for caregivers and families and impedes adequate oral care. However, few reports have assessed the current status of this modality or the measures employed to deal with related issues [14, 15].

Hence, the purpose of this study was to determine the status of implants placed in elderly individuals requiring long-term nursing and domiciliary dental care in Japan and to investigate the suitable measures for overcoming the associated limitations.

## Methods

A questionnaire was sent via mail to 1000 dentists providing domiciliary dental care within the Tokyo metropolitan area in 2017. The participants were randomly selected using a table of random numbers from registered dentists who provided domiciliary dental care. The questionnaire comprised 13 questions (Table 1) classified under the following three categories: basic information of the dentists (3 questions), actual implant status of patients requiring domiciliary dental care (5 questions), and implants in an aging society (5 questions). This study was performed after obtaining approval from the ethics committee of Nihon University School of Dentistry (No. 2016-18).

**Table 1:** Summary of the questionnaire sent to dentists who provided domiciliary dental care

Basic information of the dentists	Q1. Years of clinical experience
	Q2. Years of experience providing domiciliary dental care
	Q3. Provision of implant treatment in the respondent’s own clinic
Actual implant status of patients requiring domiciliary dental care	Q4. Type of facilities visited for domiciliary dental care and the number of implant patients
	Q5. Types or status of implant prostheses encountered in domiciliary dental care
	Q6. Types of implant-related complications encountered
	Q7. Countermeasures/treatment for implant-related problems and complications
	Q8. Oral care/care instructions provided in domiciliary dental care
Implants in an aging society	Q9. Whether there is a necessity of implants (prostheses) in patients requiring domiciliary dental care
	Q10. Indications for implants (prostheses) in domiciliary dental care
	Q11. Necessity of consulting services (from implant societies or dental associations or universities) for implant problems/complications in domiciliary dental care
	Q12. Whether implant treatment history/information provided by patients was useful

## Results

In total, 365 dentists (36.5%) responded to the questionnaire. The mean duration of clinical experience was 27.5 years (median 22.5 years), while the mean duration of experience in providing domiciliary dental care was 11 years (median 7.5 years). Of the 365 respondents, 189 (51.7%) confirmed that they performed implant treatment in their own clinics. The types of facilities visited for domiciliary dental care and the number of implant patients encountered are shown in Table 2. Six types of facilities were visited for domiciliary dental care: patients’ homes, special nursing home for the elderly (SNHs), long-term care health facilities (LCHFs), private nursing homes for the elderly (PNHs), hospitals, and day care services (DCSs) for individuals with dementia. Implant patients accounted for 2% of the total number of patients receiving domiciliary dental care. The percentage differed across facilities, being higher in homes, PNHs, and DCSs.

**Table 2:** Types of facilities visited for domiciliary dental care in the Tokyo metropolitan area and the number of implant patients in these facilities

Type of facility	Number of facilities visited	Total number of patients	Number of implant patients (percentage of total patients)
Patient’s homes	2857	3637	90 (2.5%)
Special nursing home for the elderly	410	1024	11 (1.1%)
Long-term care health facilities	60	671	8 (1.2%)
Private nursing homes for the elderly	291	170	9 (5.3%)
Hospitals	32	240	3 (1.3%)
Daycare services for individuals with dementia	6	36	1 (2.8%)
Total	3656	5878	122 (2.1%)

### Actual implant status of patients requiring domiciliary dental care

**Fig. 1:**
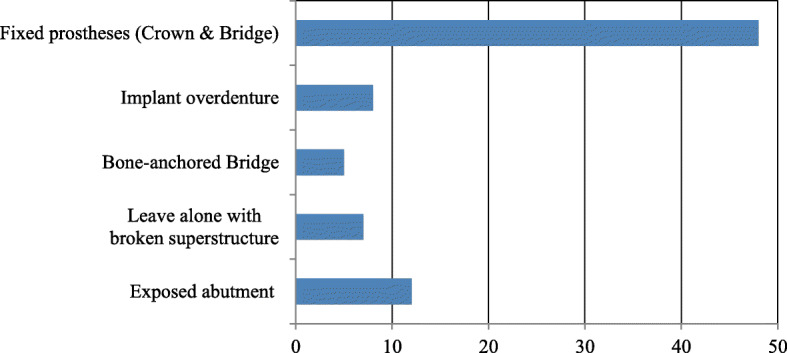
Q5. Types or status of implant prostheses encountered in domiciliary dental care (multiple answers were allowed)

**Fig. 2:**
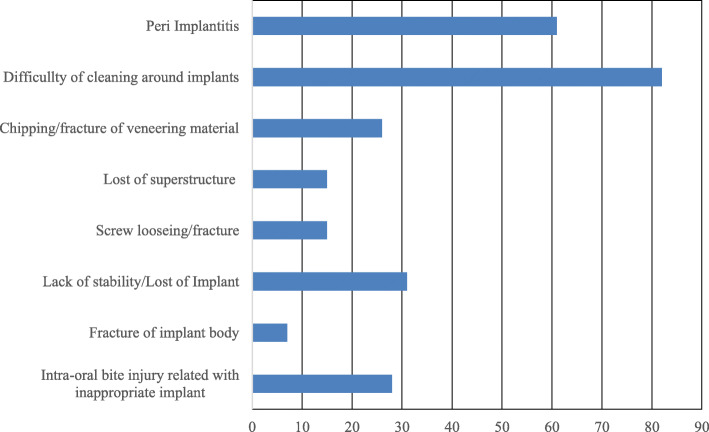
Q6. Types of implant-related complications encountered (multiple answers were allowed)

**Fig. 3:**
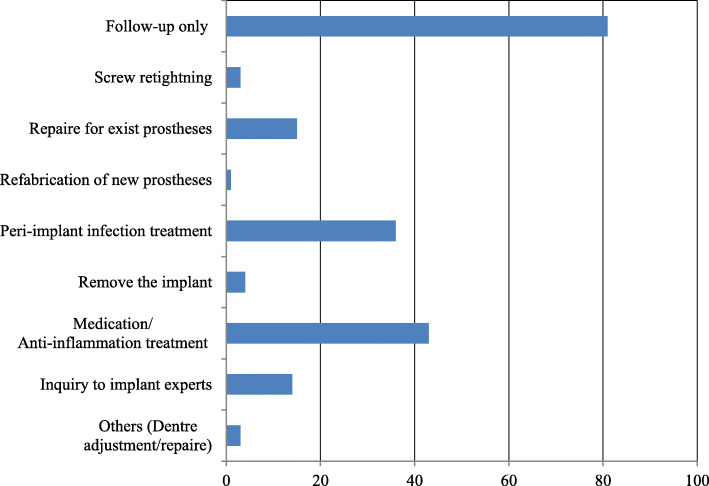
Q7. Countermeasures/treatment for implant-related problems and complications (multiple answers were allowed)

**Fig. 4:**
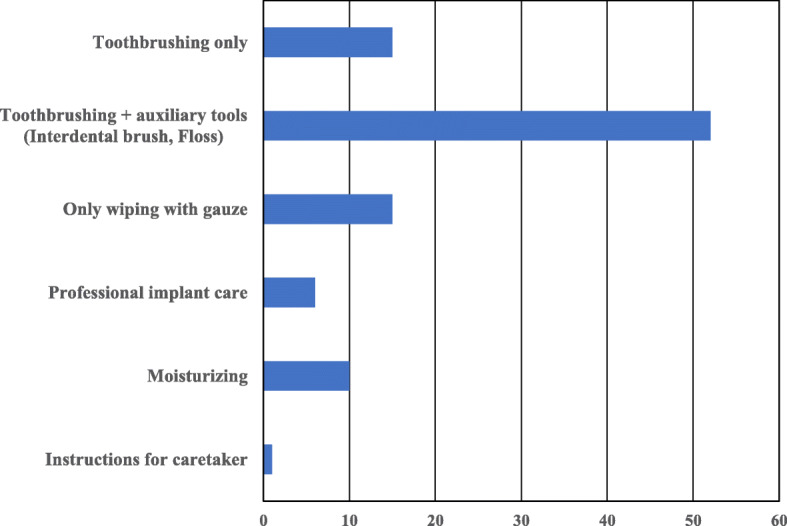
Q8. Oral care/care instructions provided in domiciliary dental care (multiple answers were allowed)

The most common implant superstructure encountered was a fixed prosthesis (crowns and bridges). Patients with exposed implant abutments and broken superstructures that were left untreated were also evaluated, and we found that these problems were difficult to manage via domiciliary dental care (Fig. 1). Many implant-related complications were associated with insufficiency or difficulty in cleaning around the implant, ultimately resulting in peri-implantitis. In addition to prosthetic complications such as chipping or fracture of veneering materials, loosening or fracture of abutment screws, and loss of cement retention, serious complications such as implant fracture or loss were also found (Fig. 2). These complications were frequently managed by routine follow-ups. Several patients underwent passive treatments, such as cleaning the area around the implant, scaling and polishing, and/or pharmacological modalities; this highlights the difficulties associated with proactive management of implants via domiciliary dental care (Fig. 3). Oral care primarily involved routine tooth-cleaning methods using equipment, such as toothbrushes, interdental brushes, and dental floss; no aggressive intervention was used. Oral hygiene instructions were provided to the families or caregivers (Fig. 4).

### Implants in an aging society

**Fig. 5:**
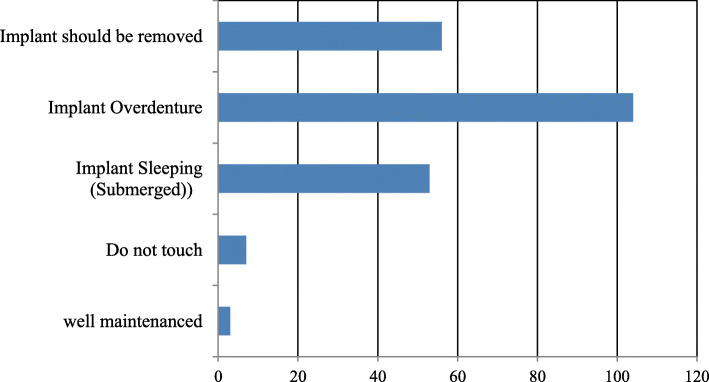
Q10. Indications for implants (prostheses) in domiciliary dental care

Regarding the need for implant treatments in patients requiring nursing care, 73% of the respondents opined that implants were not necessary in such patients. Moreover, many respondents preferred to manage implants using measures such as implant overdentures, implant removal, or conversion to sleeping (submerged) implants before patients reached the stage of requiring nursing and domiciliary dental care (Fig. 5). Additionally, 88% of the respondents stated that consulting services were necessary for implant-related problems, and 90% stated that information regarding the implant treatment (position of implant placement, implant system used, retained methods of superstructure) was necessary.

## Discussion

The response rate for the survey in this study was 36.5%, which is slightly lower than the typical response rate for postal surveys [16]. This was a fact-finding survey pertaining to implant patients requiring domiciliary dental care. Accordingly, if we assume the presence of bias due to factors such as the lack of survey completion because of no implant patients or no experience or interest in implant treatment, the actual implant status of patients requiring nursing and domiciliary dental care could be worse than that suggested by the results of this survey.

### Domiciliary dental care

This survey found that approximately 62% of patients who received domiciliary dental treatment/care resided in their homes. Various types of nursing facilities are visited for providing domiciliary dental care. SNHs are permanent residential facilities for individuals who require constant care, cannot be cared for at home, and/or have relatively severe systemic conditions, such as immobility or dementia. LCHFs are temporary residential facilities for elderly individuals requiring medical care or rehabilitation that are primarily centered on rehabilitation measures to enable the individual to return home. PNHs are residential facilities that mainly provide services of daily life, including care services (bathing, toileting, feeding), household assistance (washing, cleaning), and health and medical care. Generally, patients cover all the expenses for using PNHs. DCSs are day care facilities for patients with dementia wherein lifestyle care and functional training are provided on an outpatient basis during the day.

### Actual implant status of patients requiring domiciliary dental care

In this study, 2% of the total number of individuals receiving domiciliary dental care were implant patients. This proportion is slightly lower than the proportion of implant treatment in those aged 65 years and more (3.8%), according to the Survey of Dental Diseases conducted in Japan in 2016 [13]. With regard to the distribution of implant patients according to the type of facility, we found the highest percentage in PNHs (5.3%), followed by DCSs and homes. Elderly individuals of a relatively higher socioeconomic group reside in PNHs; consequently, the proportion of implant patients in these facilities was high. The significant number of patients showing evidence of poor hygiene maintenance around the implant, resulting in peri-implantitis, as well as the presence of serious complications such as implant body fracture or implant loss indicated that the patient or caregiver did not perform oral care in an appropriate manner. We also found that a passive approach was employed for the management of biological complications. This could be attributed to the unfamiliarity of dentists involved in domiciliary dental care with implants. Aggressive interventions and invasive treatments are difficult because of limitations in the treatment environment. Thus, difficulties in providing appropriate dental treatment via domiciliary dental care result in improperly maintained superstructures and inadequately repaired prostheses. In this study, regarding the implant superstructure (crowns and bridges), a detailed analysis of the prosthetic retention options (screw, cement) and the type of facing material was not possible. However, many mechanical complications were answered, including veneering material chipping/fracture, screw loosening/fracture, loss of retention (crown detachment), and implant body fracture associated with these types of superstructures. In addition, because of economic limitations associated with aging, the patients may not be able to afford expensive dental treatments.

Furthermore, we found that oral care around the implant primarily involved the use of toothbrushes, interdental brushes, and dental floss, accompanied by cleaning strategies, such as wiping with gauze and moisturizing. This indicates that the maintenance of cleanliness around implants was prioritized, even if aggressive treatment for peri-implantitis could not be performed. However, the patient’s family or caregiver barely received instructions regarding oral hygiene maintenance, resulting in inadequate routine oral care. As many elderly individuals depend on their families or caregivers for oral care, educational activities that will enable the caregivers to provide a certain level of oral care to dependent individuals with oral implants are necessary.

### Relationship between an aging society and implant treatment

In this study, 73% of dentists responded that implants were not necessary for patients requiring nursing care, with reasons including difficulty in providing oral care, need for invasive treatment, and difficulty in managing the prosthetic aspects of the implants. This opinion was generalized not only among dentists, but also among family members and caregivers of the patients. The participants of this study were randomly selected, and the respondents were not grouped according to their age or clinical experience, although there was a tendency for relatively experienced dentists to provide domiciliary dental care. Additionally, there was no disagreement regarding the problem of implants in an aging society and the importance/difficulty of domiciliary dental care depending on the clinical experience (age) of dentists. The long-term success of implants is dependent on regular checkups at dental clinics and routine oral hygiene maintenance. This is based on the premise that the patient is healthy and able to visit dental clinics in the long term. Accordingly, measures for the management of implant patients in the current super-aging society are essential. Müller and Schimmel [14] used the term “back-off” to advocate a shift from fixed prosthesis to a more simplified and easy-to-manage oral environment toward the end of life. This not only simplifies the provision of oral care but also prevents the build-up of biofilm and reduces the risk of aspiration pneumonia. Many respondents opined that measures such as implant-supported overdentures, implant removal, or sleeping (submerged) implants should be employed before patients reach the stage of requiring nursing and domiciliary dental care. However, if we consider the mental and financial conditions of patients, obtaining consent for changing the implant prosthesis, removing the implant, or converting the implant to a sleeping one (while the patients are still healthy) would be difficult. Furthermore, many implant patients have natural teeth as well as implant prostheses; hence, oral care for both the natural teeth and implant prostheses is required. The following factors were issues faced by domiciliary dentists/dental hygienists and caregivers: (1) little knowledge about dental implants, (2) difficulty in identifying implant-supported fixed prostheses, and (3) not familiar with special oral hygiene procedures for implants.

## Conclusions

With the limitation of low response rate to the questionnaire in this study, we found that approximately 2% of patients requiring domiciliary dental care in the Tokyo metropolitan area in Japan are implant patients; this is close to the overall percentage of implant patients in Japan. Many implants are restored using fixed prostheses, and various prosthetic and biological complications, primarily peri-implantitis, are treated using simple symptomatic measures or are left untreated. These findings suggest the necessity of simplifying the oral environment and making oral care a simple task before aging individuals require nursing and domiciliary dental care.

## Data Availability

All data generated or analyzed during this study are included in this published article.

## Abbreviations

SNHs: Special nursing home for the elderly
LCHFs: Long-term care health facilities
PNHs: Primary nursing homes
DCSs: Day care services

## References

[1] Arai H, Ouchi Y, Toba K, Endo T, Shimokado K, Tsubota K, et al. Japan as the front-runner of super-aged societies: perspectives from medicine and medical care in Japan. Geriatr Gerontol Int. 2015;15(6):673–87. 10.1111/ggi.12450. 10.1111/ggi.12450 25656311

[2] World Health Organization. Men, ageing and health: achieving health across the life span; https://www.who.int/ageing/publications/men/en/. Accessed March 20th 2021.

[3] Health and Welfare Services for the Elderly. Annual Health, Labour and Welfare Report 2017; https://www.mhlw.go.jp/english/wp/wp-hw11/index.html. Accessed February 1st 2021.

[4] Ettinger RL (2012). Dental implants in frail elderly adults: a benefit or liability?. Spec Care Dentist.

[5] Isaksson R, Becktor JP, Brown A, Laurizohn C, Isaksson S (2009). Oral health and oral implant status in edentulous patients with implant-supported dental prostheses who are receiving long-term nursing care. Gerodontology.

[6] Engfors I, Ortorp A, Jemt T (2004). Fixed implant-supported prostheses in elderly patients: a 5-year retrospective study of 133 edentulous patients older than 79 years. Clin Implant Dent Relat Res.

[7] Srinivasan M, Meyer S, Mombelli A, Müller F (2017). Dental implants in the elderly population: a systematic review and meta-analysis. Clin Oral Implants Res.

[8] Costa FO, Takenaka-Martinez S, Cota LO, Ferreira SD, Silva GL, Costa JE (2012). Peri-implant disease in subjects with and without preventive maintenance: a 5-year follow-up. J Clin Periodontol.

[9] Visser A, Hoeksema AR, de Baat C, Vissink A (2009). Treatment and subsequent care of oral implants for care-dependent patients. Ned Tijdschr Tandheelkd.

[10] Visser A, de Baat C, Hoeksema AR, Vissink A (2011). Oral implants in dependent elderly persons: blessing or burden?. Gerodontology.

[11] Sato Y, Koyama S, Ohkubo C, Ogura S, Kamijo R, Sato S, et al. A preliminary report on dental implant condition among dependent elderly based on the survey among Japanese dental practitioners. Int J Implant Dent. 2018;4(1):14. 10.1186/s40729-018-0125-7. 10.1186/s40729-018-0125-7 PMC5938220 29736592

[12] Ishimaru M, Ono S, Morita K, Matsui H, Yasunaga H (2019). Domiciliary dental care among homebound older adults: a nested case-control study in Japan. Geriatr Gerontol Int.

[13] Sato Y, Koyama S, Ohkubo C, Ogura S, Kamijo R, Sato S, et al. Dental implant care and trouble among dependent patients based on the questionnaire survey among Japanese dental practitioners. BMC Oral Health. 2020;20(1):335. 10.1186/s12903-020-01279-0. 10.1186/s12903-020-01279-0 PMC7687807 33238973

[14] Müller F, Schimmel M (2016). Revised success criteria: a vision to meet frailty and dependency in implant patients. Int J Oral Maxillofac Implants.

[15] Kimura T, Wada M, Suganami T, Miwa S, Hagiwara Y, Maeda Y (2015). Dental implant status of patients receiving long-term nursing care in Japan. Clin Implant Dent Relat Res.

[16] Kalantar JS, Talley NJ (1999). The effects of lottery incentive and length of questionnaire on health survey response rates: a randomized study. J Clin Epidemiol.

